# New North American Chrysauginae (Pyralidae) described by E.D. Cashatt

**DOI:** 10.3897/zookeys.344.5609

**Published:** 2013-10-22

**Authors:** M. Alma Solis, Everett D. Cashatt, Brian G. Scholtens

**Affiliations:** 1 MAS, Systematic Entomology Laboratory, USDA, National Museum of Natural History, P.O. Box 37012, MRC 168, Washington, DC USA 20013-7012; 2 EDC, Everett D. Cashatt, Illinois State Museum, 1011 E. Ash Street, Springfield, IL 62703, USA; 3 BGS, Brian G. Scholtens, Biology Department, College of Charleston, Charleston, SC, USA 29424

**Keywords:** Chrysauginae, Pyralidae, North America, *Campsis radicans*

## Abstract

A Ph.D. dissertation completed by E.D. Cashatt in 1968 entitled “Revision of the Chrysauginae of North America” does not meet the criteria of publication so the new taxa described therein are not available per the International Code of Zoological Nomenclature. In order to validate the taxa proposed in that document we formally describe and illustrate the following: *Arta brevivalvalis* Cashatt, **sp. n.,** *Heliades lindae* Cashatt, **sp. n.,** *Paragalasa* Cashatt, **gen. n.**, *Paragalasa exospinalis* Cashatt, **sp. n.**, and *Penthesilea sacculalis baboquivariensis* Cashatt, **subsp. n.** We summarize other taxonomic actions proposed in the dissertation and those proposed by subsequent authors. We provide the current nomenclatural status with the literature citation of the paper in which the current status was proposed. A lectotype is designated for *Clydonopteran tecomae*. Adult holotypes and associated labels, and genitalia of paratypes are newly illustrated.

## Introduction

Cashatt’s (1968) Ph.D. dissertation entitled “Revision of the Chrysauginae of North America” included numerous nomenclatural acts that are unavailable according to the most recent International Code of Zoological Nomenclature (ICZN 1999). Cashatt distributed only two copies of his dissertation (Cashatt 1968) on North American Chrysauginae: the mandatory one at Catholic University of America, and one at the library at The Natural History Museum, London, thus the entire dissertation does not constitute a published work (ICZN 1999, p. 6, Article 8). In 1969, *Dissertation Abstracts International* published and widely distributed the abstract of this dissertation and offered copies of the dissertation for sale. Whether the dissertation (Cashatt 1968) minimally meets criteria for what constitutes a published work (i.e. ICZN, Article 8, p. 6) is ambiguous (Haman and Huddleston 1980). As an example, Fletcher and Nye (1984) considered the names *Basacallis* and *Paragalasa* available stating: “Cashatt’s revision was originally a dissertation submitted to the Catholic University of America for the degree of Doctor of Philosophy. It was published on paper by University Microfilms International (UMI) and has been available since June 1969 when it was advertised for sale in *Diss. Abst. Int*. (B) 29(12): 4696.” Fletcher and Nye (1984) include these genera only because a copy was available to them at The Natural History Museum in London. The work was not accessible, however, to other institutions or scientists in the world short of visiting London or Catholic University. In contrast, Cashatt believed his dissertation to be unpublished, and he wrote a formal description of *Basacallis* in 1984. In our opinion the ICZN Recommendation (ICZN 199, p. 9, 8A) that dissemination should be in scientific journals and series, precludes the assumption of two copies constituting wide availability. In addition, the Code recommends that new names be sent to *Zoological Record*, but in this case they were not. So while the abstract published by UMI was widely available, we propose that the nomenclatural acts in the rest of the dissertation are not available.

In this work we make available by publication the taxa described in the dissertation by Cashatt (1968). We replicate the text from the dissertation with minimal editing (only as needed) from the dissertation for *Arta brevivalvalis* Cashatt, sp. n., *Heliades lindae* Cashatt, sp. n., *Paragalasa* Cashatt, gen. n., *Paragalasa exospinalis* Cashatt, sp. n., and *Penthesilea sacculalis baboquivariensis* Cashatt, subsp. n. We update the terminology of the genitalia, but not that of the wing venation. We attribute authorship of all taxa to E. D. Cashatt. We include redescriptions of the monotypic *Penthesilea* Ragonot, 1891 and *Penthesilea sacculalis sacculalis* Ragonot, 1891 because the new subspecies description would have been difficult to comprehend otherwise. We provide illustrations of the adults and genitalia (i.e., not the illustrations from the dissertation) from the type specimens located in the National Museum of Natural History, Washington, DC (USNM). We also summarize other taxonomic actions (Table 1) by Cashatt (1969) and the current status of taxa in two major taxonomic lists, the Moths of America north of Mexico (Munroe 1983), and the Atlas of Neotropical Lepidoptera (Solis et al. 1995). Cashatt (1968) used the following acronyms for collections where material is deposited: AMNH (American Museum of Natural History, New York, USA), CNC (Canadian National Collection, Ottawa, Canada), CU (=CUIC, Cornell University Insect Collection, Ithaca, New York, USA), USNM (=NMNH, National Museum of Natural History, Washington, DC, USA).

**Table 1.** Nomenclatural acts and attributions relating to taxa in Cashatt (1968). See text for more information.

**Taxon name**	**Action**	**Attribution**
*Salobrena* Walker, 1863	Revised status as genus	Cashatt (1969)
*Clydonopteron tecomae* Riley, 1880	Synonym of *Pyralis sacculana*	Miller and Becker (1989)
*Pyralis sacculana* Bosc, [1800]	Combination in *Clydonopteron* Riley	Miller and Becker (1989)
*Basacallis* Cashatt, 1984	Genus description	Cashatt (1984)
*Artopsis* Dyar, 1908	Synonym of *Parachma* Walker	Barnes and McDunnough (1917)
*Artopsis borregalis* Dyar, 1908	New revised status of *Parachma ochracealis*	Present paper
*Polloccia* Dyar, 1910	Synonym of *Acallis* Ragonot	Cashatt (1969)
*Polloccia alticolalis* Dyar, 1910	Combination in *Acallis* Ragonot	Solis et al. (1995)
*Balidarcha* Dyar, 1914	Synonym of *Anemosella* Dyar	Cashatt (1969)
*Balidarcha cuis* Dyar, 1914	Synonym of *Anemosella viridalis* B. & McD.	Munroe (1983)
*Balidarcha cuis* Dyar, 1914	Combination in *Anemosella* Dyar	Munroe (1983)
*Xantippides* Dyar, 1908	Synonym of *Arta* Grote	Cashatt (1969)
*Xantippides descansalis* Dyar, 1908	Synonym of *Arta epicoenalis* Ragonot	Munroe (1983)
*Xantippides descansalis*	Combination in *Arta* Grote	Munroe (1983)
*Acallis centralis* Dyar, 1910	New synonym of *Acallis gripalis* Hulst	Present paper
*Anemosella polingalis* B. & B., 1926	New synonym of *Anemosella basalis* Dyar	Present paper
*Xantippe beatifica* Dyar, 1921	New synonym of *Arta epicoenalis* Ragonot	Present paper
*Xantippe beatifica* Dyar, 1921	New combination in *Arta* Grote	Present paper
*Xantippe uranides* Dyar, 1921	New synonym of *Heliades mulleolella* Hulst	Present paper
*Xantippe uranides* Dyar, 1921	New combination in *Heliades* Ragonot	Present paper
*Heliades huachucalis* Haimbach, 1915	New revised status as species	Present paper
*Negalasa rubralis* B. & McD., 1913	Revised status as species	Solis et al. (1995)
*Arta brevivalvalis*	New species	Present paper
*Heliades lindae*	New species	Present paper
*Paragalasa*	New genus	Present paper
*Paragalasa exospinalis*	New species	Present paper
*Penthesilea sacculalis sacculalis*	New revised status as subspecies	Present paper
*Penthesilea sacculalis baboquivariensis*	New subspecies	Present paper

## Taxonomic actions (see Table 1)

In his abstract, Cashatt (1969) stated, “*Clydonopteron* Riley is reinstated as a genus separate from *Salobrena* Walker”; it had been synonymized by Hampson (1897). The revised status of *Salobrena* is valid and attributable to Cashatt (1969). In the dissertation, Cashatt (1968) also synonymized *Clydonopteron tecomae* Riley, 1880 as a junior synonym of *Pyralis sacculana* Bosc, [1800]. This synonymy was independently discovered and published by Miller and Becker (1989) as a new synonymy (see Landis et al. 1992); so the correct attribution of the status of *Clydonopteron tecomae* as a junior synonym of *Pyralis sacculana* is Miller and Becker (1989). Miller and Becker (1989) also newly combined *Pyralis sacculana* in *Clydonopteron*; so the correct attribution for the new combination *Clydonopteron sacculana* is Miller and Becker (1989). In the dissertation Cashatt (1968) stated he was unable to locate the type specimen of *Pyralis sacculana*, but based on illustrations he was “convinced it is conspecific with *Clydonopteron tecomae*.” He located two female types of *Clydonopteron tecomae* without locality data, but labeled “1878, USNM Type No. 366” in the USNM. He designated one of these specimens as the lectotype and the other as the paralectotype. The lectotype and paralectotype are newly designated here with attribution to Cashatt, and labeled as such in the USNM. Cashatt noted that Riley (1880) also provided a description of the life history; eggs are laid in seedpods of trumpet vine (*Campsis radicans* (L.) Seem. ex Bureau, Bignoniaceae) where larvae and pupae develop. Landis et al. (1992) published more on the biology of this species and described the egg stage. In Landis et al. (1992) EDC mistakenly used the year 1969 for the date of his dissertation in the References Cited.

Cashatt (1984) described the new genus *Basacallis* Cashatt and designated *Parachma tarachodes* Dyar, 1914, as the type species, and that is the correct attribution for availability and validity. Solis et al. (1995) correctly attributed this genus to Cashatt, but used the incorrect date of 1969, instead of 1984.

In the dissertation Cashatt (1968) synonymized the following genera and transferred and/or synonymized type species. *Artopsis* Dyar, 1908 was synonymized with *Parachma* Walker, [1866] in the checklist (Cashatt 1968), and it did not appear in the abstract (Cashatt 1969). Attribution for this synonymy should be Barnes and McDunnough (1917) where it first appeared, although it was not stated explicitly. Cashatt (1968) synonymized the type species, *Artopsis borregalis* Dyar, 1908 with *Parachma ochracealis* Walker, [1866], and published this new synonymy in Cashatt (1984), but Solis et al. (1995) revised the status of *Artopsis borregalis*, considering it a valid species. Herein we synonymize *Artopsis borregalis* with *Parachma ochracealis*, revised status, and attribute this action to Cashatt. In addition, Cashatt (1968) listed 1909 as the date of publication for *Artopsis* and *Artopsis borregalis* which should have been 1908, but the confusion of the year is due to the publication of volume 10 of the Proceedings of the Entomological Society of Washington in two different years; issues 1 and 2 (pp. 1–118) in 1908 and issues 3 and 4 (pp. 119–221) in 1909.

Cashatt (1968) synonymized *Polloccia* [misspelled in the dissertation as *Pollocia*] Dyar, 1910 with *Acallis* Ragonot, 1891 and transferred the type species, *Polloccia alticolalis* Dyar, 1910 to *Acallis*. The abstract (Cashatt 1969) mentions this generic synonymy so the correct attribution for the status of *Polloccia* as a junior synonym of *Acallis* is Cashatt (1969). The abstract (Cashatt 1969) did not transfer the type species, but Solis et al. (1995) newly combined *Polloccia alticolalis* in *Acallis*, so Solis et al. (1995) is the correct attribution.

Cashatt (1968) synonymized *Balidarcha* Dyar, 1914 with *Anemosella* Dyar, 1914 and the generic synonymy does appear in the abstract (Cashatt 1969), so the correct attribution of the status of *Balidarcha* as a junior synonym of *Anemosella* is Cashatt (1969). Cashatt (1968) synonymized *Balidarcha cuis*, Dyar, 1914 with *Anemosella viridalis* Barnes & McDunnough, 1912. The abstract (Cashatt 1969) did not transfer or synonymize the type species, but Munroe (1983) treated *Balidarcha cuis* as a new synonym, and as a new combination although not stated, so the correct attribution for the status of *Balidarcha cuis* as a junior synonym of *Anemosella viridalis* and a new combination in *Anemosella* is Munroe (1983). *Balidarcha cuis* was also treated as a synonym by Solis et al. (1995).

Cashatt (1968) synonymized *Xantippides* Dyar, 1908 with *Arta* Grote, 1875 and transferred the type species, *Xantippides descansalis* Dyar, 1908 as a synonym of *Arta epicoenalis* Ragonot, 1891. The generic synonymy appears in the abstract (Cashatt 1969), so the correct attribution of the status of *Xantippides* as a junior synonym of *Arta* is Cashatt (1969). The transfer of the type species does not appear in the abstract (Cashatt 1969). Munroe (1983) published the synonymy of *Xantippides descansalis* as a new combination and new synonymy, so the correct attribution for the status of *Xantippides descansalis* as a new combination and junior synonym of *Arta epicoenalis* is Munroe (1983).

Cashatt (1968) synonymized the following species in his dissertation, but they do not appear in the abstract (Cashatt 1969) so we newly synonymize these species below. Cashatt (1968) synonymized *Acallis centralis* Dyar, 1910 with *Acallis gripalis* Hulst, 1886. Munroe (1983) and Solis et al. (1995) treated *Acallis centralis* as a valid species of *Acallis*. Herein we synonymize *Acallis centralis* with *Acallis gripalis*, new synonymy, with attribution to Cashatt.

Cashatt (1968) synonymized *Anemosella polingalis* [misspelled in the dissertation as *pollingalis*], Barnes and Benjamin 1926 with *Anemosella basalis* Dyar, 1914. It was treated as a valid species by Munroe (1983) and Solis et al. (1995). Herein we synonymize *Anemosella polingalis* with *Anemosella basalis*, new synonymy, with attribution to Cashatt.

Cashatt (1968) synonymized and combined *Xantippe beatifica* Dyar, 1921 with *Arta epicoenalis* Ragonot, 1891. It was treated as a valid species of *Xantippe* by Munroe (1983) and Solis et al. (1995). Herein we synonymize *Xantippe beatifica* with *Arta epicoenalis*, new combination, new synonymy, with attribution to Cashatt.

Cashatt (1968) synonymized and combined *Xantippe uranides* Dyar, 1921 with *Heliades mulleolella* (Hulst, 1887). It was treated as a valid species in *Xantippe* in Munroe (1983) and Solis et al. (1995). Herein we synonymize *Xantippe uranides* with *Heliades mulleolella*, new combination, new synonymy, with attribution to Cashatt.

Haimbach (1915) described *Pyrausta huachucalis*, but McDunnough (1939) revised its status as a junior synonym of *Heliades mulleolella* Hulst, 1887. Munroe (1983) and Solis et al. (1995) also treated *Heliades huachucalis* as a junior synonym of *Heliades mulleolella*. Cashatt (1968) elevated *Heliades huachucalis* to species stating: “…this western species is distinct from the eastern one.” Herein we elevate *Heliades huachucalis* to species status, revised status,with attribution to Cashatt.

*Negalasa rubralis* Barnes & McDunnough, 1913 was treated as a subspecies of *Negalasa fumalis* by Munroe (1983) but was not clearly stated to be a revised status; it is historically interesting to note that Cashatt (1968) did the same thing. *Negalasa rubralis* was elevated back to species by Solis et al. (1995), but again it was not clearly stated to be a revised status. Due to the ambiguity of its status, we are leaving this status as determined by Solis et al. (1995).

## Taxon descriptions

### Arta brevivalvalis

Cashatt sp. n.

http://zoobank.org/BC60CF21-AF03-4025-B272-251827BA2B87

http://species-id.net/wiki/Arta_brevivalvalis

Figs 1 , 5–7

#### Description.

**Head.** Labial palpus reddish-brown laterad, inner surface ochreous; frons and vertex light reddish-brown to purplish; occiput tan to ochreous.

**Thorax.** Upper surface reddish-brown to tan, under surface darker. Forewing reddish- to purplish-brown with ochreous antemedial and postmedial lines; antemedial line irregular and extending obliquely from two-thirds costa to nearly one-half hind margin; postmedial line irregular and directed slightly inward near costa, extending from three-fifths costa to near anal angle; distance between the two lines greater at costa than at hind margin; fringe ochreous; under surface brown, purplish-red near costa and outer margin. Hind wing grayish-brown with ochreous fringe; underside purplish-red near costa and outer margin, a short postmedial line from costa fading inward. Legs purplish-brown with mid femur, midtibia, and inner surface of hind leg ochreous.

**Abdomen.** Ochreous dorsad, reddish-brown ventrad, terminal fringe ochreous.

**Male genitalia.** Uncus broad and shovel-shaped; tegumen narrow, pedunculus unmodified; vinculum broad, saccus not narrowly produced anteriad as in *Arta statalis*, but rounded; juxta acutely hooked dorsad near base with apex directed slightly dorsad; valva as in *Arta statalis* except shorter with a broader base, apex unidentate; phallus long and slender with apex flattened without a coecum or cornutus.

**Female genitalia.** Ovipositor moderately enlongate, papillae anales small and unilobate; posterior apophysis extremely short; anterior apophysis short as in *Arta statalis*; ostium bursae wide; lamella antevaginalis broad and V-shaped, opening near anterior margin of eighth sternite; bursa copulatrix simple with inception of ductus seminalis below antrum; without a signum.

#### Type data.

The type specimens are located as noted below. The male holotype is from Palmerlee, Arizona (no other data given) and is labeled as the holotype. Fifty-three male and twenty-two female paratypes from UNITED STATES: ARIZONA are labeled as follows: two females, Catalina Mts., no date given, Oslar Coll. (USNM); two males and two females, Catalina Mts., June 10, 1903, Oslar, Coll. (USNM); one female, Huachuca Mts., (USNM); one male, Madera Canyon, Santa Rita Mts., Aug. 18, 1953, Robert J. Ford (CNC); one male and one female, Madera Canyon, Santa Rita Mts., Aug. 19, 1953. Robert J. Ford (CNC); one male, Madera Canyon, Santa Rita Mts., Aug. 9, 1953 (CNC); one male, Palmerlee, Sept. 8–15 (USNM); twenty-five males and nine females, Palmerlee, no date given (USNM); seven males and four females, Ramsey Canyon, Huachuca Mts., Sept. 1-2, 1927, J. C. Bradley Coll. (CU); ten males and two females, White Mts., elevation 7200 ft., Aug. 1–15, 1925, Poling Coll. (USNM); one male and one female, White Mts., elevation 7200–11500 ft., Aug. 10–30, 1925, O. C. Poling (USNM); three males, White Mts., Apache Co., near McNary P. O., Sept. 15–30, 1925, O. C. Poling.

#### Life history.

Unknown.

#### Remarks.

It is difficult to separate *Arta brevivalvalis* from *Arta statalis* and *Arta epicoenalis* on the basis of maculation. The ochreous fringe is sometimes a useful diagnostic character but is not reliable. The distance between the antemedial and postmedial lines is variable.

An examination of the genitalia is necessary for accurate identification. The flattened and spade-shaped uncus of *Arta brevivalvalis* easily separates this species from *Arta statalis* and *Arta olivalis* that have a narrow and more cylindrical shape. The valva of *Arta olivalis* is long and slim. The uncus of *Arta epicoenalis* is flattened, but not constricted at the base as in this species. The ostium bursae of *Arta brevivalvalis* is broad compared to that of *Arta statalis*, and the anterior apophyses are extremely short. The anterior apophyses of *Arta olivalis* and *Arta epicoenalis* are absent.

**Figures 1–4.**
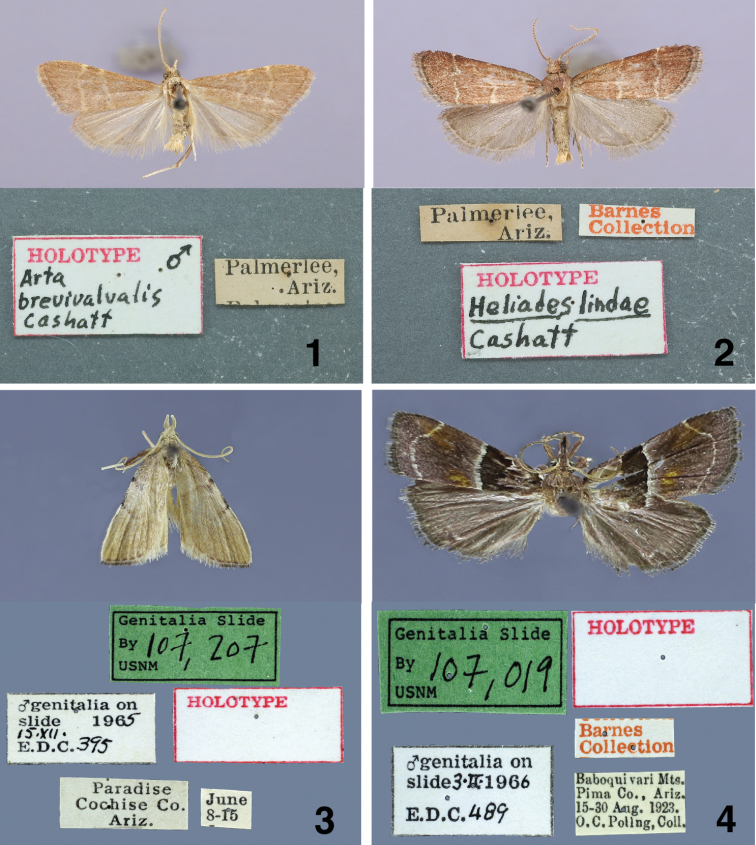
Male holotypes of adults and labels. **1** *Arta brevivalvalis* **2** *Heliades lindae* **3** *Paragalasa exospinalis* **4** *Penthesilea sacculalis baboquivariensis*.

**Figures 5–10.**
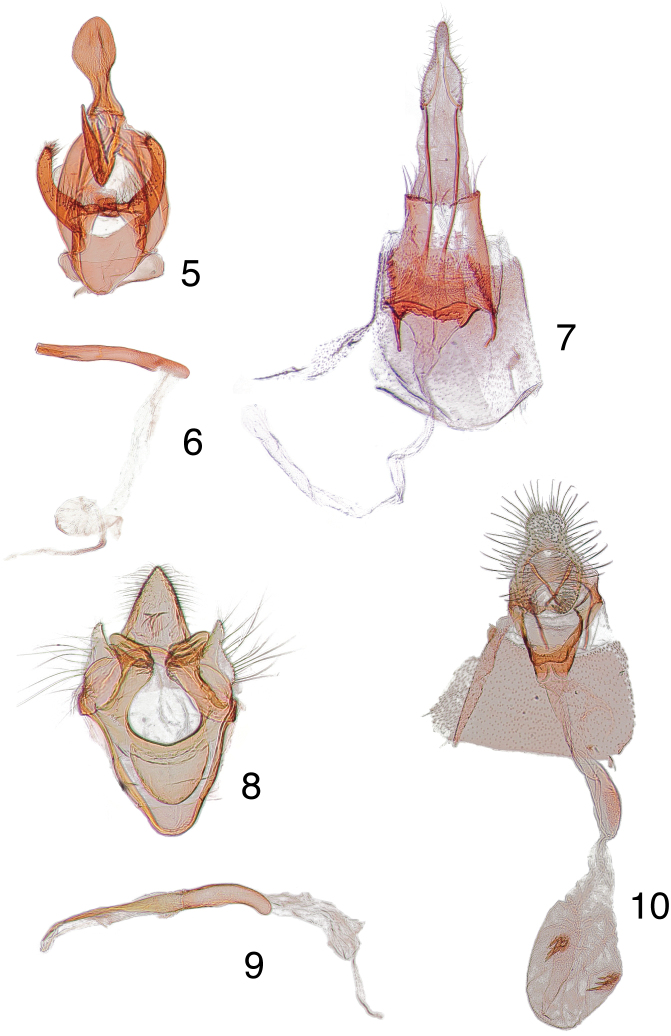
Male, female genitalia. **5** *Arta brevivalvalis* paratype male, USA, Arizona, Palmerlee, [no collection date on label], EDC 981, USNM 104473**6** phallus, data same as previous **7** paratype female, USA, Arizona, Palmerlee, [no collection date on label], EDC 982, USNM 104474 **8** *Heliades lindae* paratype male, USA, Arizona, Palmerlee, [no collection date on label], EDC 80, USNM 105993 **9** phallus, data same as previous **10** paratype female, USA, Arizona, Palmerlee, [no collection date on label], EDC 84, USNM 104482.

### Heliades lindae

Cashatt sp. n.

http://zoobank.org/F91B3D1C-706B-4341-80C4-10AA5117497D

http://species-id.net/wiki/Heliades_lindae

Figs 2 , 8–10

#### Description.

**Alar expanse.** 15 to 17 mm.

**Head.** Labial palpus dark reddish-brown with fuscous on under surface; frons, vertex, occiput, and antenna brownish-red.

**Thorax.** Upper surface brownish-red; under surface fuscous. Forewing brownish-red with white dentate antemedial and postmedial lines; antemedial line extending from about two-fifths costa to nearly two-fifths inner margin, postmedial line extending from three-fourths costa to just proximad of anal angle; terminal line fuscous; fringe gray with a dark medial line; under surface grayish-brown with apex brownish-red. Hind wing light grayish-brown; fringe gray with a dark medial line, under surface gray with apex reddish-brown. Legs fuscous with midtibia and tarsus white.

**Abdomen.** Upper surface concolorous with hind wings; terminal fringe ochreous.

**Male genitalia.** Uncus long and aculeate, setose dorsad; tegumen narrow dorsad; vinculum broad with a well-developed saccus, but more broadly rounded; gnathos reduced to a slender arm articulating at base of uncus; valva with sacculus small and papilliform, setose; valva heavily sclerotized and plate-shaped with apex truncate, ankylosed with flat truncate tips of arms produced by juxta; juxta shield-shaped and ankylosed with inner margin of vinculum; phallus long and slender, coecum well-developed.

**Female genitalia.** Ovipositor extremely short; apex of papillae anales bilobate and broad; eighth segment extremely short; anterior apophysis about one-half length of posterior apophysis; opening of ostium bursae at anterior of eighth sternite, small and sclerotized; anterior margin of sinus vaginalis bilobate and more broadly joined to the anterior margin of the eighth sternite; inception of ductus seminalis below antrum; ductus bursae weakly sclerotized and constricted near junction of corpus bursae; signum a pair of spines.

#### Type data.

All the type specimens are in the USNM. The male holotype is from Palmerlee, Arizona (no other data given) and is labeled as the holotype. Twenty-three male and nineteen female paratypes from UNITED STATES: ARIZONA are labeled as follows: one female, Baboquivari Mts., Pima Co., 1-15 Sept. 1923, O. C. Poling; one male, Chiricahua Mts., July 4, H. G. Hubbard; one male, Fort Grant, July 20, H. G. Hubbard; one female, Hereford, no date, C. R. Biedermann; one male, Huachuca Mts., no date; one female, Huachuca Mts., Aug. 8-15; one male, Madera Canyon, Santa Rita Mts., Aug. 19, 1953, Robert J. Ford; one male, Nogales, July 15, 1903, Oslar; one female, Oracle, July 28, 1924, E. P. Van Duzee; nine males, four females, Palmerlee, no date given; one female, Palmerlee, Cochise Co., Aug. 1–7; one male, one female, Paradise, Cochise Co., no date; four males, five females, Paradise, Cochise Co., July; two females, Paradise, Cochise Co., Aug.; one female, Paradise, Cochise Co., Aug. 1–7; one male, S.W.R.R., 5 mi. W. Portal, Cochise Co., 5400 ft., July 9, 1956, Cazier and Ordway; one female, Santa Catalina Mts., no date given; one male, White Mts., El. 7000 ft., July 15–22, 1925, O. C. Poling.

#### Life history.

Unknown.

#### Remarks.

It gives me pleasure to name this species in honor of my wife, Linda. The coloration of *Heliades lindae* is similar to *Heliades huachucalis* except the former is lighter and more reddish. The antemedial and postmedial lines of *Heliades huachucalis* are white, but unlike *Heliades lindae* the lines are margined with fuscous.

### Paragalasa

Cashatt gen. n.

http://zoobank.org/E96A1722-5429-4294-9CCC-8A2727C56859

http://species-id.net/wiki/Paragalasa

Figs 3 , 11–13

Paragalasa Cashatt, 1968, *nomen nudum*, Solis et al. 1995

#### Type species.

*Paragalasa exospinalis*, Cashatt, new species.

#### Description.

**Head.** Labial palpus porrect, length approximately equal to head width; maxillary palpus vestigial, two segmented, pilifers moderately developed; proboscis well developed; frons rounded with a tuft produced obliquely; vertex and occiput roughly scaled; ocellus immediately posteriad to base of antenna; chaetosema a row of fine setae along ocular sutura posteriad to ocellus.

**Thorax.** Forewing long and narrow, costa slightly incurved near middle, apex sub-lanceolate, outer margin rounded; sexually dimorphic: male with a small glandular vesicle at base of costa, discal cell shorter than in female, R_1_ not reaching costa, posterior angle obtuse; female without a glandular vesicle, R_1_ intercepting the costa, posterior angle of discal cell acute; both sexes with Sc long, R_1_ arising from just before end of discal cell; R_2_ stalked short with R_3_, R_4_, and R_5_, stem arising from anterior angle of discal cell; R_3_ stalked with R_4_ and R_5_; R_4_ and R_5_ coincident; M_1_ separate, arising from anterior angle of discal cell; male with M_2_ separate, M_3_ end Cu_1_ stalked short; Cu_2_ separate, arising from posterior angle of discal cell; female M_2_ and M_3_ stalked short, Cu_1_ and Cu_2_ widely separated; 2A and 3A separate at base, anastomosed briefly a short distance from base; retinaculum normally developed. Hind wing frenulum normal; Sc and Rs anastomosed beyond end of discal cell; M_1_ separate from anterior angle of discal cell; M_2_ and M_3_ short stalked from posterior angle of discal cell; posterior angle of discal cell extremely long and slender; Cu_1_ and Cu_2_ widely separated. Legs long, midtibia with two scale tufts.

**Abdomen.** Long and slender, without scale tufts.

**Mala genitalia.** Uncus moderately broad with apex rounded, slender arms from base modified to articulate with gnathos; tegumen narrow dorsad; pedunculus strongly modified for articulation with gnathal arms; vinculum moderately broad with saccus slightly produced; gnathos slender and aculeate, apex hooked dorsad; valva with sacculus distinct from valva, ventral margin of sacculus rounded; transtilla weak and incomplete; juxta with dorsal margin V-shaped; phallus small, coecum long, apex with microspines, cornutus with spines short and spur-shaped.

**Female genitalia.** Ovipositor moderately long; papillae anales apex unilobate; anterior apophysis slightly longer than posterior apophysis; lamella postvaginalis triangulate; ostium bursae membranous, antrum lightly sclerotized, inception of ductus seminalis just below antrum; ductus bursae extremely long; corpus bursae small and without a signum.

#### Remarks.

The venation and genitalia indicate a close relationship between this genus and *Negalasa*. The male *Paragalasa* has a small glandular vesicle at the base of the costa on the forewing, but is without a costal spur. The costa is straight. *Negalasa* and *Galasa* have a larger glandular vesicle, an incurved costal margin and a costal spur at the end of Sc. The uncus of *Negalasa* is more narrow and pointed, the tip of the valva is directed acutely mediad, and the phallus has a broadly rounded coecum and cornutus with long spines. The male genitalia of *Paragalasa* is similar to *Galasa* except the dorsal margin is V-shaped, there is no process on the sacculus, and the phallus is smaller with a long cylindrical coecum and a small cornutus. The female *Paragalasa* has the inception of the ductus seminalis just below the antrum. The ductus bursae is extremely long with a small corpus bursae. *Negalasa* has the inception of the ductus seminalis more sclerotized, nearly two-thirds length from ostium bursae, and a large corpus bursae. The female venation of *Paragalasa* and *Negalasa* is identical. The male forewing of *Negalasa* shows more specialized structures.

**Figures 11–16.**
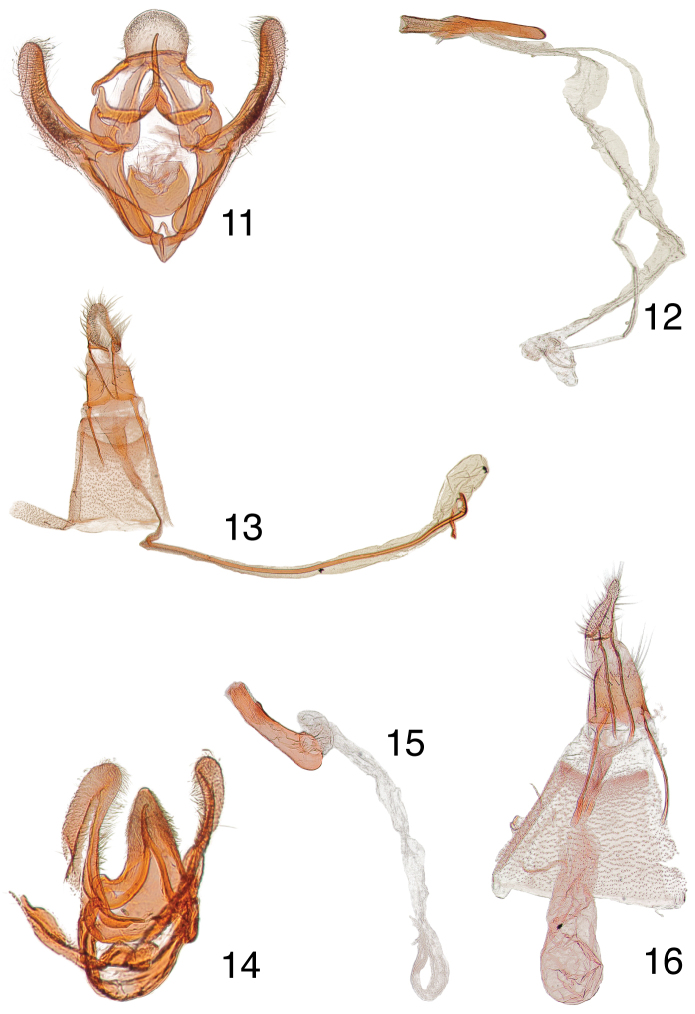
Male, female genitalia. **11** *Paragalasa exospinalis* holotype male, USA, Arizona, Cochise Co., Paradise, June 8-15 [no year given], EDC 395, USNM 107207 **12** phallus, data same as previous **13** paratype female, USA, Arizona, Redington, [no collection date on label], EDC 398, USNM 107089 **14** *Penthesilea sacculalis baboquivariensis* holotype male, USA, Arizona, Pima Co., Baboquivari Mts., 15–30 Aug 1923, O.C. Poling, Coll., Barnes Collection, EDC 489, USNM 107019 **15** phallus, data same as previous **16** allotype female, USA, Arizona, Pima Co., Baboquivari Mts., [days crossed out] Aug 1924, O.C. Poling, Coll., Barnes Collection, EDC 138, USNM 100018.

### Paragalasa exospinalis

Cashatt sp. n.

http://zoobank.org/8B654928-5F29-4939-A494-E15FB480B90A

http://species-id.net/wiki/Paragalasa_exospinalis

Figs 3 , 11–13

Paragalasa exospinalis Cashatt, 1968, *nomen nudum*, Solis et al. 1995

#### Description.

**Alar expanse.** 19 to 22 mm.

**Head.** Labial palpus ochreous, darker laterad; frons, vertex, and occiput ochreous to tan; antenna ochreous.

#### Thorax.

Upper surface pale reddish-brown, lower surface reddish-brown. Forewing pale reddish-ochreous; costa irrorated with fuscous, especially at base and at origin of antemedial and postmedial lines; antemedial line light reddish-brown, indistinct, extending from about one-third length of costa to about one-third length of inner margin; postmedial light reddish-brown and extending from about two-thirds length of costa sharply excurved to about two-thirds length of inner margin. Hind wing light pinkish to brownish-white with terminal line darker; fringe ochreous to brownish-ochreous. Legs ochreous, sprinkled with dark brown laterad, midtibia scale tufts fuscous.

**Abdomen.** Upper surface greyish-ochreous, fuscous laterad, lower surface ochreous.

**Genitalia.** As described for the genus.

#### Type data.

All the type specimens in the USNM. The male holotype is from Paradise, Cochise Co., Arizona, June 8–15 and is labeled as the holotype. Twenty-six male and thirteen female paratypes from UNITED STATES: ARIZONA are labeled as follows: UNITED STATES: ARIZONA: eighteen males and two females, Baboquivari Mts., Pima Co., Ariz., elevation approximately 5000 ft., 15–30 June, 1923, O. C. Poling Coll.; four males, Brown’s Canyon, Baboquivari Mts., Pima Co., Ariz., elevation approximately 5000 ft., 1–15 June 1923, O. C. Poling Coll.; one female, Brown’s Canyon, Baboquivari Mts., Pima Co., Ariz., elevation approximately 5000 ft., 15–30 May 1923, O. C. Poling Coll.; two females, Huachuca Mts., Ariz., no date given; three males and three females, Palmerlee, Arizona, no date given; one male, Paradise, Cochise Co., Ariz., June 8–15; one male, Paradise. Cochise Co., Ariz., July; one male and two females, Redington, Ariz., no date given; one female, Santa Rita Mts., Ariz., June 11, 1898. B. A. Schwarz.

#### Life history.

Unknown.

#### Remarks.

This species might be confused with *Negalasa rubralis* at first glance. Distinguishing characters are the fuscous antemedial and postmedial lines on the costa, generally lighter coloration, and the longer, more narrow forewings. The distinctness of the median band is variable.

### Penthesilea

Ragonot, 1891

http://species-id.net/wiki/Penthesilea

Figs 4 , 14–16

Penthesilea Ragonot, 1891: 493.

#### Type species.

*Penthesilea sacculalis* Ragonot, by monotypy.

#### Description.

**Head.** Labial palpus decumbent; length of male palpus nearly equal to head width, length of female palpus longer than head width; maxillary palpus vestigial; proboscis moderately well-developed; frons rounded with vestiture extended obliquely; vertex smooth-scaled; occiput rough-scaled; eye large; ocellus separated from base of antenna by scales; without a chaetosema.

**Thorax.** Forewing broad and arched at base, apex broadly rounded, outer margin and anal angle broadly rounded; sexually dimorphic; male with a tympanic vesicle at base of costa, with a large hair-pencil gland as in *Salobrena*; female without a glandular vesicle; both sexes with Sc long, intercepting costa past one-half length; R_1_ and R_2_ separate; R_3_ and R_4_ stalked, R_5_ from stem; M_1_ from end of discal cell just below anterior angle; M_2_ and M_3_ separate and arising from posterior angle of discal cell; Cu_1_ and Cu_2_ separate and arising from below posterior angle of cell; 1A absent, 2A and 3A separate at base but briefly anastomosed a short distance distad; retinaculum of male loop-shaped and strongly developed with inner surface corrugated as in *Salobrena*, *Clydonopteron*, *Satole* and *Tosale*. Hind wing of male with frenulum stoutly developed with a short hook at base, female normal; Sc arched at base and anastomosed with Rs past end of discal cell; M_1_ arising from anterior angle of discal cell; M_2_ and M_3_ separate, arising from the posterior angle of discal cell; Cu_1_ and Cu_2_ separate, from before the posterior angle of the discal cell. Legs with scale tufts on mid and hind tibia.

**Abdomen.** Short and stout; male with a small lateral pleurite on the terminal segment bearing a tuft of scales as in *Tosale* and *Salobrena*.

**Male genitalia.** Uncus narrow, dorsally setose, aculeate with apex rounded, base with arms produced for articulation with gnathos, tips broadly rounded; vinculum narrow, saccus not produced anteriad; gnathos apex aculeate, gently curved dorsad, arms gradually expanded to broad articulation with modified pedunculus and base of uncus; valva narrow, tips directed slightly upward and mediad, sacculus without a clasping process; transtilla moderately developed and incomplete; juxta trapezoidal, dorsal margin concave; phallus slightly curved upward, proximal end slightly expanded, coecum small, without a cornutus.

**Female genitalia.** Ovipositor moderately short, apex of papillae anales unilobate; anterior apophysis slightly longer than posterior apophysis; lamella postvaginalis triangulate; anterior margin of eighth tergite rounded; ostium bursae membranous; a sclerotized constriction below antrum on ductus seminalis as in *Tosale*; inception of ductus seminalis at junction of ductus bursae and corpus bursae; corpus bursae without a signum.

#### Remarks.

The genera *Penthesilea* and *Tosale* are closely related. The female genitalia have a membranous ostium bursae, a short sclerotized constriction on the ductus bursae, and the inception of the ductus seminalis at the junction of the ductus bursae and ostium bursae are common to both genera. *Tosale* differs in having the anterior margin of the eighth tergite heavily sclerotized. The male genitalia show more divergence. The uncus and valva of *Penthesilea* are more narrow than in *Tosale* and the saccus is not produced. Both genera have small lateral pleurites on the hind margin of the last abdominal segment of the male for support of lateral scale tufts. The venation indicates the *Tosale* is more specialized, with stalking of R_2_, R_3_, R_4_, and R_5_ in the forewing. The forewing of *Penthesilea* has R_2_ free with R_3_ and R_4_ stalked and R_5_ short-stalked. Both genera have M_1_ widely separately from the stem of R_5_.

### Penthesilea sacculalis sacculalis

Ragonot, 1891, revised status

http://species-id.net/wiki/Penthesilea_sacculalis_sacculalis

Penthesilea sacculalis Ragonot, 1891: 493.

#### Description.

**Alar expanse.** 13 to 16 mm.

**Head.** Labial palpus dark brown with black; frons and vertex dark brown with white around the base of scape; occiput reddish-brown.

**Thorax.** Brown dorsad and ventrad. Forewing dark brown to fuscus; basal angle occasionally overscaled with reddish-brown, base darker than distal part; antemedial line white and slightly excurved; a yellow suffusion distad of white antemedial line; postmedial line white with a large brownish-orange suffusion near the costa, acutely excurved mediad; fringe fuscous. Hind wing dark brown to fuscous; Cu_2_ with a small white spot near outer margin, a small reddish-brown dash along Cu_2_ anteriad and posteriad to spot; fringe fuscous. Legs dark brown to fuscous; midtarsi white, hind tarsi white except first subsegment fuscous.

**Abdomen.** Brown overscaled with fuscous and reddish-brown, lateral tufts fuscous.

**Genitalia.** As described for genus.

#### Type data.

One male holotype, with no locality data is in the Museum National D’Histoire Naturalle in Paris.

#### Material examined.

Six males and eleven females from the following localities:

UNITED STATES: FLORIDA: Coconut Grove (USNM); Lake Placid, Archbold Bio. Sta., May (USNM); Royal Palm State Park (USNM); Winer Park [Winter Park?] (AMNH). GEORGIA: Atlanta (USNM). LOUISIANA: Lafayette, June (AMNH). NORTH CAROLINA: Southern Pines, July, Aug. (USNM). TEXAS: Brownsville (USNM); San Benito, July, Sept. (USNM). VIRGINIA: Skyland, July (USNM).

**Life history.** Unknown.

#### Remarks.

Ragonot (1891) states that the type specimen is probably from North America. Of the specimens that I have examined, it more nearly matches the specimens from Florida. The specimens from Florida are darker and smaller than those from Louisiana, Texas, Georgia, and North Carolina.

### Penthesilea sacculalis baboquivariensis

Cashatt subsp. n.

http://species-id.net/wiki/Penthesilea_sacculalis_baboquivariensis

Figs 4 , 14–16

#### Description.

**Alar expanse.** 13 to 17 mm.

**Head.** Labial palpus dark pinkish-brown; frons, vertex, occiput, and antenna pinkish-brown.

**Thorax.** Upper and lower surfaces pinkish-brown. Forewing same as the nominate species except pinkish-brown. Hind wing pinkish-brown occasionally with a small white spot as in *Penthesilea sacculalis*, but with no dark scaling anteriad or posteriad. Legs dark pinkish-brown with mid and hind tarsi white.

**Abdomen.** Pinkish-brown with terminal scale tufts pinkish-brown.

**Genitalia.** As described for the genus.

#### Type data.

The holotype, allotype, and forty-four paratypes are from the Baboquivari Mts., Pima Co., Arizona. The male holotype and female allotype are in the USNM. The male holotype is from Baboquivari Mts., Pima Co., Arizona, August 15-30, 1923, O. C. Poling, is labeled as the holotype, and the female allotype is labeled, August, 1924, O. C. Poling. The paratypes are labeled as follows: UNITED STATES: ARIZONA: two males and two females, elevation approximately 5000 ft., June 15-30, 1923 (USNM); one female, July 1–15, 1924, O. C. Poling (USNM); one female, July 15–30, 1924, O. C. Poling (USNM); two females, Aug. 1–15, 1924, O. C. Poling (USNM); one male and four females, Aug. 15–30, 1923, O. C. Poling (USNM); one male Aug., (USNM); three males and ten females, Sept. 1-15, 1923, 1924; O. C. Poling (USNM); one male and one female, Sept. 15–30, 1924, O. C. Poling (USNM); one male and four females, Oct. 1–15, 1923, O. C. Poling (USNM); one male and one female, Oct. 15–30, 1924, O. C. Poling (USNM); three males and two females, Sabino Canyon, Sept. 5-6, 1951, L. M. Martin (CNC); one male and one female, Sabino Canyon, Sept. 5, 1951, R. J. Ford (CNC).

#### Life history.

Unknown.

#### Remarks.

This subspecies differs from the nominal species only by the pinkish-brown coloration.

## Supplementary Material

XML Treatment for Arta brevivalvalis

XML Treatment for Heliades lindae

XML Treatment for Paragalasa

XML Treatment for Paragalasa exospinalis

XML Treatment for Penthesilea

XML Treatment for Penthesilea sacculalis sacculalis

XML Treatment for Penthesilea sacculalis baboquivariensis
